# A mitochondrion-like ribosome in an insect endosymbiont

**DOI:** 10.64898/2026.10.18.759926

**Published:** 2026-10-22

**Authors:** Israel S Fernandez, Jessica Warren, Justs Nwachukwu, Dewight Williams, Amaia Gonzalez-Magana, Noa Spencer, John McCutcheon

## Abstract

Understanding how the mitochondrion evolved from a bacterial endosymbiont to an organelle is obscured by time and a lack of comparative examples. It is clear that mitochondrial ribosomes are derived from bacterial ribosomes, but it is unclear whether the baroque and idiosyncratic structural changes seen in mitochondrial ribosomes are specifically driven by mitochondrial evolution or simply reflect the syndrome of being a long-term endosymbiont. Here we show that two of the most characteristic features of mitochondrial ribosomes--the acquisition of host proteins and alterations to specific structural regions--are shared by Hodgkinia, an ancient bacterial endosymbiont of cicadas. These results imply that many of the striking changes observed in mitochondrial ribosomes result from their common ancestry as bacterial endosymbionts rather than their uniqueness as mitochondria.

